# Commissioning of the Iseult CEA 11.7 T whole-body MRI: current status, gradient–magnet interaction tests and first imaging experience

**DOI:** 10.1007/s10334-023-01063-5

**Published:** 2023-01-30

**Authors:** Nicolas Boulant, Lionel Quettier, the Iseult Consortium, G. Aubert, A. Amadon, J. Belorgey, C. Berriaud, C. Bonnelye, Ph. Bredy, E. Chazel, G. Dilasser, O. Dubois, E. Giacomini, G. Gilgrass, V. Gras, Q. Guihard, V. Jannot, F. P. Juster, H. Lannou, F. Leprêtre, C. Lerman, C. Le Ster, M. Luong, F. Mauconduit, F. Molinié, F. Nunio, L. Scola, A. Sinanna, R. Touzery, P. Védrine, A. Vignaud

**Affiliations:** 1 Université Paris-Saclay, CEA, CNRS, BAOBAB, NeuroSpin, Gif Sur Yvette, France; 2 Université Paris-Saclay, CEA, Irfu, Département des Accélérateurs, de la Cryogénie et du Magnétisme, Gif Sur Yvette, France

**Keywords:** MRI, Ultra-high field, Gradient-magnet interactions

## Abstract

**Objectives:**

The Iseult MRI is an actively shielded whole-body magnet providing a homogeneous and stable magnetic field of 11.7 T. After nearly 20 years of research and development, the magnet successfully reached its target field strength for the first time in 2019. This article reviews its commissioning status, the gradient–magnet interaction test results and first imaging experience.

**Materials and methods:**

Vibration, acoustics, power deposition in the He bath, and field monitoring measurements were carried out. Magnet safety system was tested against outer magnetic perturbations, and calibrated to define a safe operation of the gradient coil. First measurements using parallel transmission were also performed on an ex-vivo brain to mitigate the RF field inhomogeneity effect.

**Results:**

Acoustics measurements show promising results with sound pressure levels slightly above the enforced limits only at certain frequency intervals. Vibrations of the gradient coil revealed a linear trend with the B_0_ field only in the worst case. Field monitoring revealed some resonances at some frequencies that are still under investigation.

**Discussion:**

Gradient-magnet interaction tests at up to 11.7 T are concluded. The scanner is now kept permanently at field and the final calibrations are on-going to pave the road towards the first acquisitions on volunteers.

## Introduction

MRI at ultra-high field (UHF) is a promising technology to explore the human brain at the mesoscopic scale and with unprecedented details enabled by the supra-linear gain in signal-to-noise (SNR) and contrast-to-noise ratio (CNR) with field strength [1–9]. The first UHF magnet was an 8 T 800 mm bore system developed by Magnex Scientific Limited for Ohio State University in 1998 [10]. This magnet was closely followed by a 7 T, 90 cm warm bore magnet that was installed at the University of Minnesota in 1999. Over an 18-year period, between 1997 and 2015 approximately 72 UHF magnets were designed and built by Magnex Scientific from their Oxfordshire factory in the United Kingdom. After many developments and efforts made by the MR community and scanner manufacturers to unleash their potential, 7 T Whole-Body (WB) MRI are now FDA and CE certified machines. With this history in mind, around 2000 it was thus barely any surprise that designing and building a WB 11.7 T MRI machine was considered unrealistic. Today a 10.5 T MRI scanner at the University of Minnesota is operational and is the largest magnetic field to date that has been used for scanning human subjects [11]. Two 11.7 T passively shielded (requiring more than 700 tons of iron shielding) MRI scanners (ASG, Genoa, Italy) with 68 cm bore size, operating at 2.3 K, are also being commissioned at NIH (Bethesda, MD, USA) and Gachon University (South Korea). The CEA with its expertise on magnets dedicated to fusion reactors and particle detectors accepted the challenge of building a WB 11.7 T magnet for MRI. And after a feasibility study, the Iseult-Inumac project was funded [12]. After nearly twenty years of research and development, prototyping, manufacturing and commissioning, first images were finally successfully obtained in 2021.

After describing the architecture of the Iseult magnet and its specifications, this article reviews the last commissioning and validation steps leading to its first images. Gradient–magnet interactions at 11.7 T were uncharted territory and were investigated gradually. On the one hand, this approach was used to minimize risks regarding the magnet but also to learn as much as possible about the behavior of the system that could have an impact on image quality. The numerous tests led to a plethora of data that one may never be able to fully understand. And many surprises occurred and led to more interrogations. « The suspense is terrible… I hope it will last» wrote Oscar Wilde. This quotation expresses what some of the team scientists felt during these years who were torn between the idea of moving forward to acquire the first images and the excitement of discovering new things, asking to be understood and investigated more deeply, along the way.

## History of the Iseult-Inumac project

Early 2000s, the CEA launched a program to develop and build a “human brain explorer”. At that time, it was the first WB-size MRI scanner project for operation at 11.7 T. The magnet was part of a larger endeavor to develop Molecular Imaging at Ultra-High Field financed through a French-German initiative involving academic (CEA and Julich research center), industrial (Siemens, Bruker, Guerbet and GE Power, by then Alstom MSA) and governmental organizations across both countries (AII, then Oséo and BPI for France, BMBF for Germany). The project was officially endorsed in April 2004 by French President Jacques Chirac and German Chancelor Gerhard Schröder. Due to the complexity of this unique MRI system and the associated technical challenges to be addressed, 5 years of extensive R&D efforts and prototyping activities were required to define the final design and validate the technical choices used to design the magnet [13–16]. After seven years of fabrication at Belfort by GE Power (Ex-Alstom) [12], the Iseult magnet was delivered to CEA in June 2017, its connection with the cryogenic plant and all the ancillary equipment was completed in October 2018. After 4 months of cooldown and another 4 months of tests, the Iseult magnet reached the field of 11.72 T for the first time on July 18th 2019 [17].

## Magnet design

**Fig. 1:**
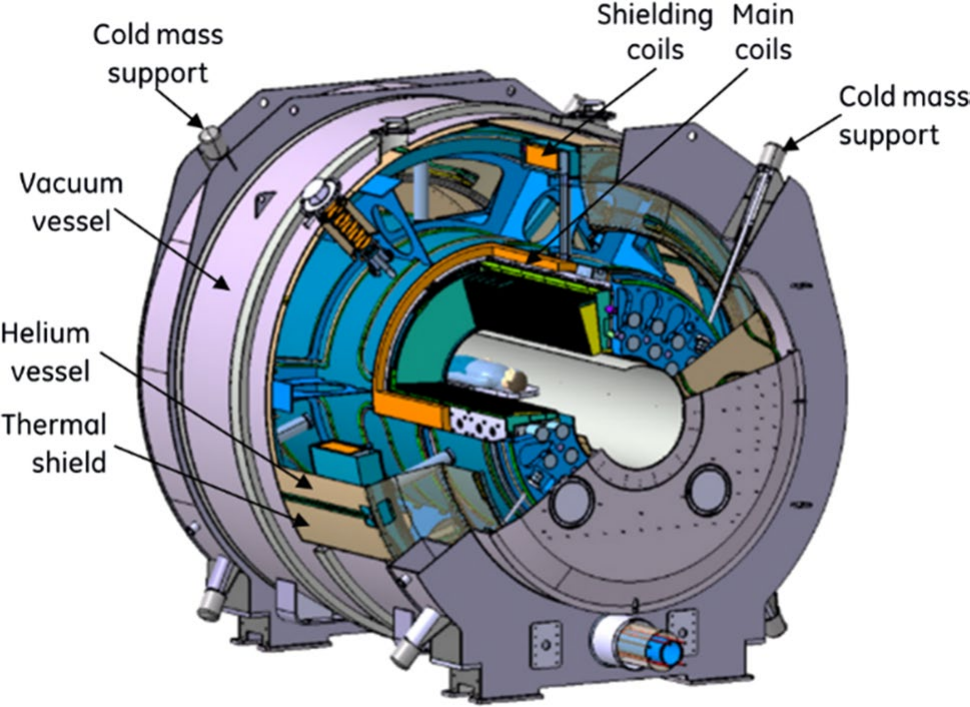
View of the 11.7 T Iseult magnet and cryostat

The core part of the Iseult MRI is an actively shielded NbTi magnet cooled with a superfluid He bath at 1.8 K, providing a homogeneous magnetic field of 11.7 T within a 90 cm warm bore operated in driven mode (Fig. 1).

Selection of the warm bore diameter or aperture is a trade-off between field homogeneity, gradient coil performance and challenges to the magnet design. 11.7 T MRI magnets are the highest-field magnets that allow the use of NbTi coils. While it would be possible to use an hybrid design that employs both Nb_3_Sn and NbTi coils in a He bath at 4.2 K, it was decided at the beginning of the design phase not to use Nb_3_Sn due to the risk of failure (Nb_3_Sn is very brittle, and the superconducting properties can only be obtained after a very complex heat treatment at high temperature during the manufacturing stage) and the prohibitive cost of the material. Even if a lot of progress has been made for the last 20 years on the usage of Nb_3_Sn, it is not clear whether the developments would outweigh the constraints and difficulties this material would impose on such a strong magnet. The most important criteria for the NbTi choice at the beginning of the project thus were the price and the maturity of the technology. Now in 2023, major progress has been made in High-temperature superconductor (HTS) technology over the last 5 years thanks to developments mostly made in fusion, thereby reducing the gap in price between NbTi and HTS strategies.

Iseult to date is the highest-field large bore scanner with active shielding, although this design decision caused the increase of the magnet size and complexity. Iseult is installed within a cylindrical hall made of a concrete tube of 10 m in diameter and 15 m in length. The estimated mass for a passive shield around the magnet was about 750 tons or even about 2000 tons if the iron plate had to be fixed on the experimental wall, which was not acceptable for the NeuroSpin building [13, 14]. The final magnet specifications are given in Table 1.

**Table 1:** Main parameters of the Iseult magnet

Item	Quantity
Current	1470 A
Operating temperature	1.8 K
Central field	11.72 T
Inductance	308 H
Stored energy	338 MJ
Mass	132 tons
Field homogeneity	< 0.5 ppm (peak to peak on 22 cm DSV)
Field stability	0.05 ppm/h

A dedicated cryogenic plant installed in the basement of the NeuroSpin building is used to cooldown the magnet at 1.8 K. The superfluid He is serviced by a separate cryogenic facility forming an integral part of the installation. During the cryogenic plant commissioning and the cooling phase, extensive tests were carried out to measure the heat loads and the cryo-mechanical performance of the cryogenic satellite connected to the He refrigerator, including the 1.8 K stage [13–15]. Last but not least, one unique aspect of the Iseult setup is the high availability strategy established to allow continuous MR exploitation throughout the year. This implies redundancy of the most critical components in case of sensor faults or failures (power supplies, Magnet Safety System, cryogenic equipment, etc.).

### Quench protection

As the main coil magnet is designed to be cryostable [16], a local quench cannot theoretically propagate. Obviously, this does not mean that a quench is impossible, e.g., resulting from a heat overload from interactions with the gradient system which could go above the Gorter-Mellink limitation where He super-fluidity vanishes and thus provides less heat dissipation [18]. Quench propagation in this case becomes very difficult to model. Therefore, as a safety measure, some magnet protection mechanism had to be implemented in the event of a quench. The complexity of the overall Iseult magnet operation, taking into account electrical and cryogenic requirements for field stability, magnet and patient safety, led us to design a Magnet Control System (MCS) and a Magnet Safety System (MSS) relying on a high availability programmable logic controller [16, 19]. MCS is permanently monitoring and controlling more than 300 devices located in the magnet cryostat and in its cryogenic and electrical ancillary (programmable logic controllers, valves, current transducers, temperature and pressure sensors). This kind of MSS design is commonly used for particle detector magnets but its use for MRI is an entirely new concept. It is based on the detection of voltages created across the superconducting coils in case of quench, and a dump resistor to dissipate the stored energy. Finally, the MSS reliability is based on voting redundancy (two out of three (2oo3) Logic) to reinforce its reliability against fault scenario detection [19]. More than 1300 fault tests and required corrective actions have been completed which allows the magnet to be kept at field without permanent on-site supervision.

### Field stability in non-persistent mode

With the exception of the Iseult magnet, all UHF magnets currently installed operate in persistent mode, where the power supply can be disconnected from the magnet after the required current has been set in the superconducting coils. The total number of joints between the different double pancakes in the magnet is about 250. With the single strand conductors used for conventional MRI magnets, superconducting joints can be used. However, with our multi-strand wire magnet this operation becomes highly risky, as about 1700 superconducting joints would be required to join the individual strands of the 170 double pancake wires to ensure the current balance in each wire. The multi-strand strategy was chosen to reduce the inductance of the main superconducting coil and thus reduce the maximum voltage induced in case of quench. As the quench protection design required, anyway, that current leads are permanently connected to a dump resistor, it was considered to use resistive joints and to keep the power supply permanently connected to the magnet.

**Fig. 2:**
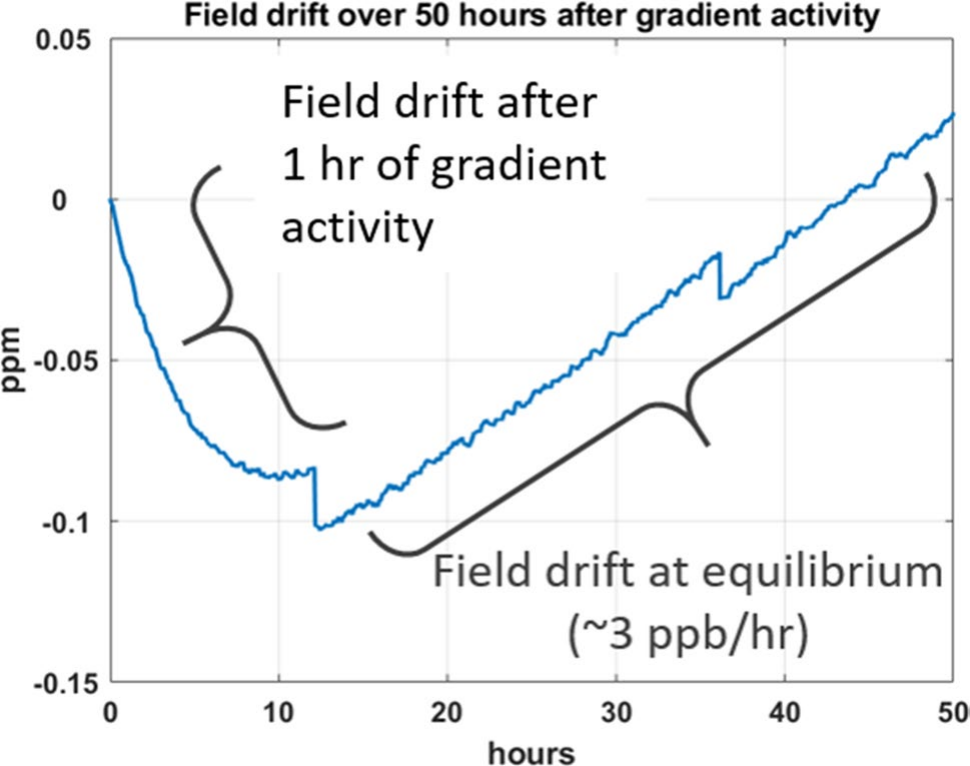
Field stability measured at 11.7 T (in ppm) with a Skope field camera for 50 h. A 1-h diffusion sequence was run before the start of the measurement. The sudden drops visible on the plot correspond to the discharge of the External Interference Shield (described in the next section)

To ensure the required field stability (better than 0.05 ppm/h) a hybrid operation mode, so-called semi-persistent mode, was developed and tested using a resistive filter in series with a fault current limiter [20], to compensate for the lack of stability of commercially available power supplies (0.1 ppm/h at best). This solution was tested successfully on a 1.5 T prototype magnet (initially built to demonstrate the innovative double pancake winding technique), as well on in a 8 T facility available at CEA Saclay [20]. First tests performed in November 2020 had already validated this field stabilization technique. The temporal field stability was finally measured at 11.7 T with a Skope clip-on field camera (Skope MRT, Zürich, Switzerland). After several iterations to adjust the current powering the fault limiter, as shown in Fig. 2 a drift of about 3 ppb/h was obtained at thermal equilibrium, well below the specification of 0.05 ppm/h. When driven out of thermal equilibrium due to gradient activity (e.g. with heating of the iron shims or the gradient coil), the drift can be affected. The same figure likewise reports for the first ~ 10 h the field drift when returning to equilibrium, after a 1-h diffusion sequence was run, still below the 0.05 ppm/h specification.

### Protection against external magnetic field interferences

**Fig. 3:**
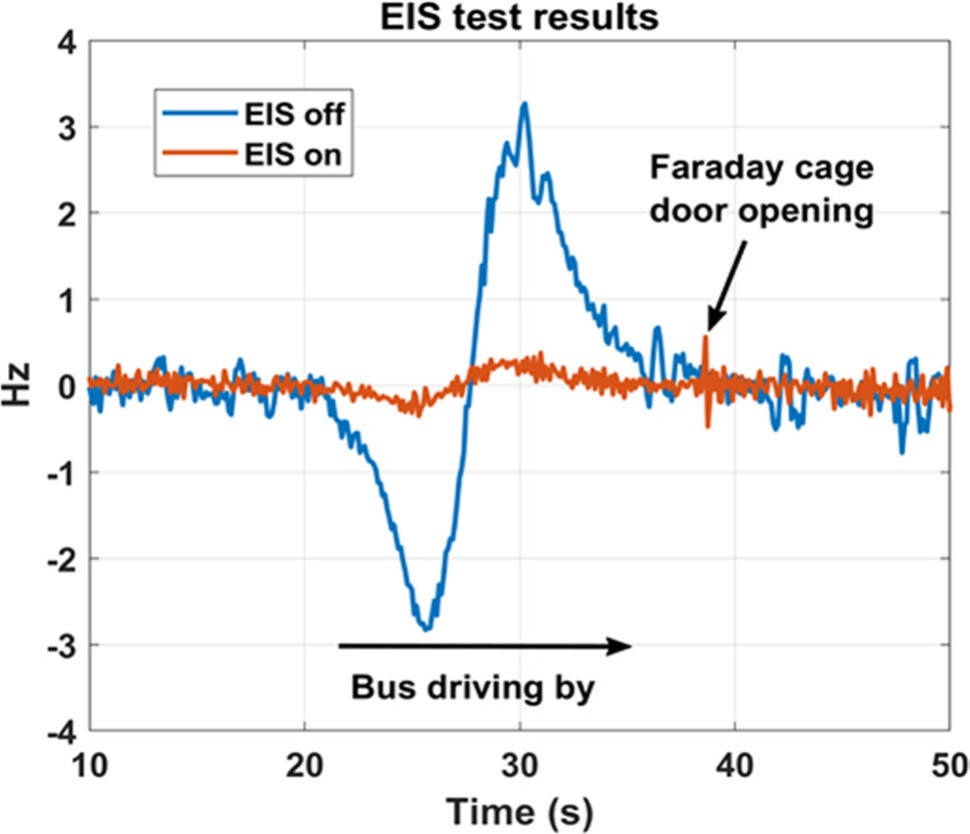
External interference shield (EIS) efficiency results characterized with a Skope field camera. Temporal resolution of the measurements was 105 ms

The magnetic field inside the central bore can be affected by external magnetic field perturbations not directly generated by the MR imaging components, including outside of the Faraday cage. They can be caused for instance by trains, buses, trucks or even elevators in the vicinity of the magnet and they can result in an unacceptable central field disturbance, or even a modification of the magnetic field quality in the useful area. The Iseult magnet is equipped with an External Interference Shield (EIS) designed to screen these external perturbations [14–16]. Extensive tests were performed to validate its operation and adjust the settings without impacting the MSS operation while the EIS circuit is discharged, or in case of failure. As illustrated in Fig. 3, the most critical perturbations were engendered with a single-decker bus driving on the road nearby (~ 15 m distance from isocenter), with the Iseult magnet at 7 T. The data clearly demonstrates the effectiveness of the EIS. With the EIS turned off, the bus engenders a ± 3 Hz field perturbation. Turning on the EIS reduces it to about ± 0.2 Hz.

### Field homogeneity

The magnet is designed to cancel up to 14 degrees of the Spherical Harmonic Expansion (SHE) of the magnetic field [16]. This is done by optimization of the axial distance between each of the 170 double pancakes constituting the main coil. The formulation given by (1) has been used for the SHE.

1 $${B}_{z}\left(r,\theta ,\varphi \right)={B}_{0}\left\{1+\sum_{n=1}^{\infty }{\left(\frac{r}{{r}_{0}}\right)}^{n}\left[{Z}_{n}{P}_{n}\left(\mathrm{cos}\theta \right)\right.\right.+\left.\sum_{m=1}^{n}\left({X}_{n}^{m}\mathrm{cos}m\varphi +{Y}_{n}^{m}\mathrm{sin}m\varphi \right){W}_{n}^{m}{P}_{n}^{m}\left(\mathrm{cos}\theta \right)]\right\},$$

where *r*_*0*_ is the reference radius and *W*_*n*_^*m*^ is a weight factor used to have comparable contributions for *X*_*n*_^*m*^, *Y*_*n*_^*m*^, *Z*_*n*_ coefficients. It is given by

2 $$W_{n}^{m} = \frac{{\left( {n - m + 1} \right)!!}}{{\left( {n + m - 1} \right)!!}}.$$

The magnetic field map in the useful imaging area was measured at different field levels with a field camera developed for the Iseult project by the Swiss company Metrolab (Metrolab, Geneva, Switzerland). The device is composed of 40 NMR probes that once sequentially rotated at 36 angular positions allow to reconstruct the field SHE with a very good accuracy and reproducibility. Shimming was initially foreseen using an active cryoshim made of several superconducting coils embedded inside the magnet cold mass and a passive device located inside the magnet bore at room temperature that can hold up to 5904 iron shim pieces [14]. However, during the MSS tests, we observed that voltages across the main superconducting coils could appear in case of a power failure of the cryoshim. These voltages are caused by the magnetic coupling between the cryoshim winding and the main magnet. MSS thresholds in this case could be exceeded, which would trigger a fast discharge of the magnet. As a consequence, it was decided to adjust the field homogeneity of the magnet using only the iron shim. We typically overconstrain the iron shim optimization problem by fitting 324 coefficients out of 1440 field values to reconstruct up to the 17th order of the SHE.

The computation of a shimming configuration is a linear optimization problem under linear constraints. Converging to a configuration that validates the homogeneity criterion still requires several steps for at least two reasons. First, there are initial uncertainties in evaluating the matrix of linear constraints due to imprecisions in the positioning of the shim pieces and unknowns in the characteristic of their ferromagnetic response. Secondly, the linear program yields a continuous solution that usually contains ambiguous values for some shimming slots (that are neither fully iron nor empty) so that the configuration practically implemented needs to be adjusted and verified. The bare magnet homogeneity at 11.7 T was 119 ppm (peak–peak) and after 8 iterations of the passive shim assembly (optimization of iron piece locations), the field homogeneity now is 1.3 ppm (peak–peak over a 22 cm diameter sphere) for a total iron mass of 30 kg. Measured SHE coefficients are given in Table 2. Although the field homogeneity does not exactly fulfill the specification of 0.5 ppm, this value is considered acceptable for now. Further improvements of the shimming capability and additional shimming iterations will be performed in the coming months to further improve field homogeneity and imaging quality.

**Table 2:** Measured SHE coefficients (in ppm of the central field) for a radius of 0.11 m

SHE coefficient	300 K	80 K 0 T	1.8 K 0 T	1.8 K–11.72 T Bare magnet	1.8 K–11.72 T Final shimming
Z1	− 132	− 42	− 5	9	− 0.22
Z2	− 105	− 61	− 15	− 17	0.19
Z3	20	9	2	2	− 0.05
X11	− 1	− 8	22	24	− 0.01
X21	59	− 2	2	2	0.15
X22	−	1	0	− 1	− 0,00
Y11	−	62	60	62	− 0.08
Y21	−	1	− 6	− 7	− 0.33
Y22	−	− 1	− 1	− 1	− 0.00

### Magnet operation summary

The magnet has now been kept permanently at 1.8 K for more than three and a half years, and at field for more than 10 months in total. The magnet has already been ramped up and down more than 10 times, with a charging/discharging time of only 5 h. As discussed previously, the high availability system has been fully commissioned. The field stability is 0.003 ppm/h, while the field homogeneity currently is 1.3 ppm (peak–peak). This value is considered as acceptable for the coming months but one more iteration will be needed later. Overall, the Iseult main magnet is now fully operational and ready for imaging.

## Gradient–magnet interaction tests

The Iseult MR system is currently equipped with the SC72 gradient coil (maximum gradient strength and slew rate of 70 mT/m and 200 T/m/s respectively, weight = 900 kg, length = 1.59 m, inner/outer diameter = 64/81 cm) designed and manufactured by Siemens Healthineers (Siemens Healthcare, Erlangen, Germany). After the cool-down of the magnet at 1.8 K, numerous tests and reaching the 11.7 T field strength, the commissioning of the Iseult MRI entered a gradient coil-magnet interaction test campaign, spread over a two-year period. Gradient coils operate under oscillating currents which generate eddy currents in the different metallic shells (e.g. He vessel, thermal shields, cryostat and lead tube) via the imperfectly shielded potential vector of the time-dependent magnetic field and which, under a constant and strong magnetic field, induce important forces and vibrations. The latter can be the source of image artefacts as well as hardware damage. Importantly, vibrations also are mostly responsible for electric fields induced in the cryostat thereby generating power deposition by Joule’s effect. If not well characterized and understood, their consequences can be disastrous and lead to magnet quench. The objective of the tests thus was twofold: (1) to determine operating modes in terms of gradient frequency, strength and duration to provide safe and optimal MR exploitation, (2) gather invaluable information to troubleshoot the system if problems (artefacts) are encountered during imaging.

The tests covered acoustics, vibrations, power deposition in the He bath, MSS voltage and field monitoring measurements. Because preventing a magnet quench was the priority, measurements were first performed with a lead tube [21] surrounding the gradient, whose goal was to absorb energy through vibro-electromagnetic coupling and thus protect the magnet. The modeling which led to the lead tube strategy could be performed only on the *Z*-axis [21] thanks to the axial symmetry. It took into account the electromagnetic fields generated by the gradient coil, the eddy currents generated in the different conductive layers (lead tube, bore tube, thermal shields, and He vessel) as well as their induced vibrations. A lot of material could be borrowed from the theory of vibrating thin shells from NASA [22]. But while a lot of literature deals with determining free vibrations and eigenfrequencies, very few address the problem of forced oscillations. Mechanical coupling between the different layers turned out to be too complex to model and therefore was first ignored, hoping that current decoupling approaches would make the non-touching concentric shells assumption reasonably valid. After discovering some imaging artefacts, and after thorough analysis and further modeling, the lead tube was removed but only after the measured power depositions in the cryostat were confirmed reasonable and under control. This section describes the results of these tests, with the two scenarios, i.e. with and without lead tube, when relevant.

### Power deposition in the He bath

The main superconducting coil operates at 1.8 K (pumped saturated bath), where helium becomes superfluid. The threshold defined to protect the Iseult magnet is 1.95 K, whereby if this temperature is reached, a slow discharge of the magnet is triggered. The 7000 L of the bath provide a 200 W h margin to operate the gradients for MRI. The cryogenic facility absorbs the natural heat losses due to radiation and conduction with current leads, and can compensate for an additional 15 W. Beyond that power, the temperature of the bath increases. But with its large enthalpy, the temperature rise is slow and with proper monitoring, MR sequences can be stopped to instantly cease power depositions induced by the gradient coils.

**Fig. 4:**
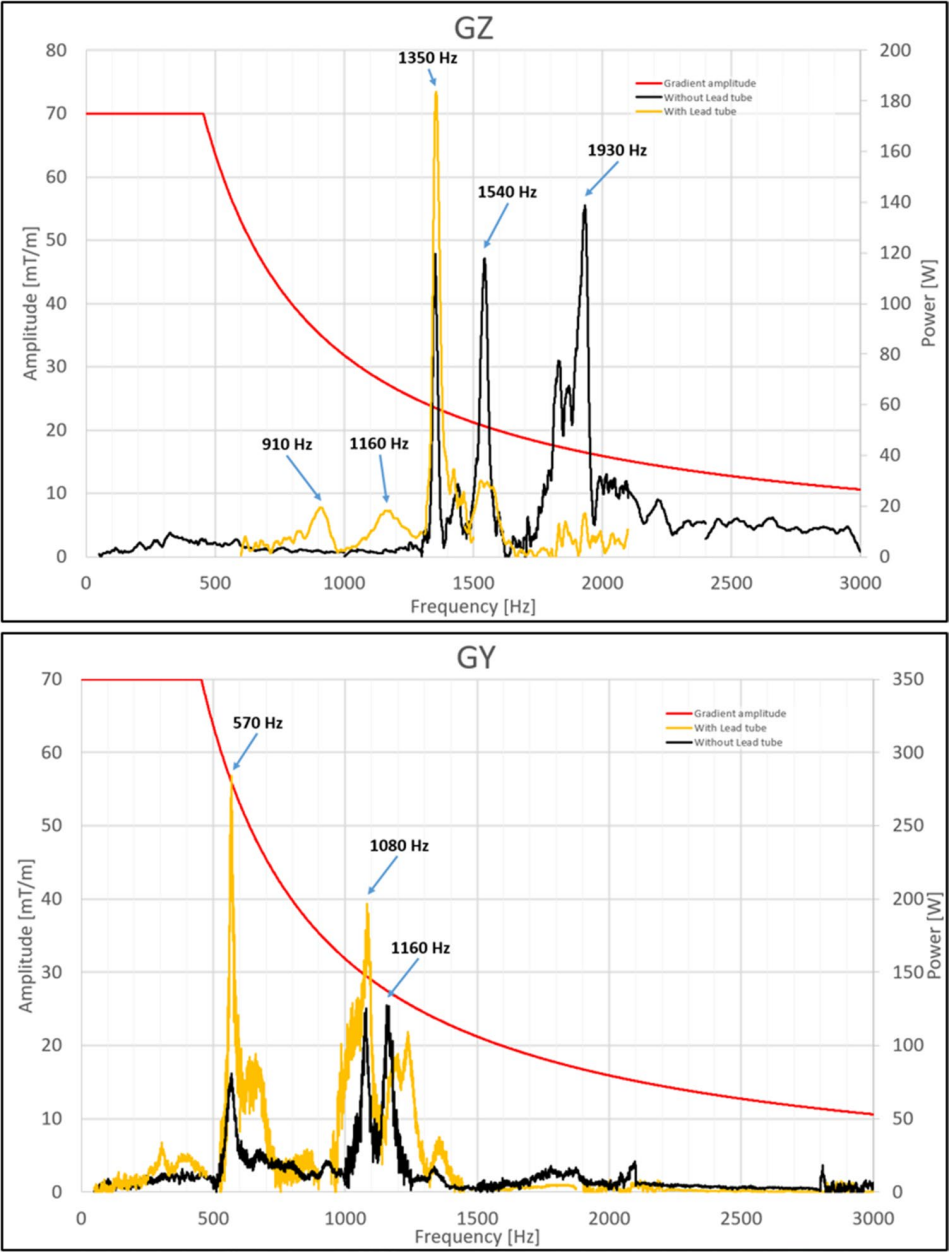
Power deposition versus gradient frequency. Results are normalized with respect to maximum gradient strength compatible with slew-rate (red curve). Top: *Z*-axis, bottom: *Y*-axis. The lead tube and no lead tube configurations correspond, respectively, to the lines in yellow and black

On most of the MR systems, He boil-off can be measured for instance with flowmeters connected within the magnet cryogenic circuit and relatively fast gradient frequency sweeps at atmospheric pressure. The Iseult cryogenic setup prevents from using this method. As a result, a new methodology had to be developed. It consists of keeping the pumping unit speed constant, which leads to a constant volume flow. An additional power deposition due to gradient activity then is compensated by less power dissipated by an electrical heater, located in the helium reservoir, to keep the sum constant. From these measurements, it then becomes possible to deduce the power deposition due to gradient activity from the pumping unit speed. While the large volume of helium and the 1.8 K temperature provide a large safety buffer for exploitation, on the other hand it requires time consuming gradient frequency sweeps (< 100 Hz/h) to cover the 0–3 kHz range and obtain the desired helium boil-off spectra. After first tests at 7 T and 10.2 T, measurements were performed at 11.7 T to yield the results presented in Fig. 4 for the *Z*- and *Y*-axes of the gradient coil (result on the *X*-axis was relatively similar to the one obtained on the *Y*-axis). The subplot for the *Z*-axis confirms the theoretical prediction with an overall less energetic spectrum with the lead tube. An important peak yet remains at 1350 Hz and is more important in the lead tube scenario. This resonance is not reproduced in the model, whose frequency is known to correspond to a mechanical resonance of the gradient (see dedicated sub-section). It is thus believed to originate from a mechanical coupling between the gradient and cryostat, not taken into account in the model. The result lead tube versus no lead tube is reversed for the *Y*-axis (i.e. more power deposition with lead tube). But again, we cannot predict at this stage the impact of mechanical coupling between the gradient and the cryostat and thus rule out an opposite diagnosis if proper mechanical decoupling was achieved.

### Magnet safety system

As explained above, the magnet safety system (MSS) is based on voltages measured continuously across the main superconducting coils. In case of quench, the detection of voltages created by resistive areas developed in the superconducting coils leads to a fast discharge of the magnet. The magnet is disconnected from its power supply using a mechanical switch, and the energy stored inside the magnet is then dissipated into an external dump resistor. A detailed model of the quench propagation and of the magnet protection was developed during the design phase. Results showed that a 1 V threshold for 750 ms would define a safe margin for the magnet (with a maximum hot spot temperature of 132 K and a maximum voltage of 3400 V in the worst fault scenario) while providing room to maneuver in the MR exploitation. If the thresholds defined above are exceeded, the system again triggers a fast, controlled, discharge of the magnet to prevent it from quenching. During frequency sweeps, voltages often exceeded the 1 V threshold. Their time scales, however, were short and never longer than 10 ms. While the cryogenic tests are very long due to the large volume of the He bath, MSS voltage measurements are fast and 0–3 kHz spectra can be acquired in a few minutes depending on the targeted accuracy. Because both measurements appear strongly influenced by vibrations, this method could turn out to be very valuable to quickly detect dangerous frequency zones. The correlation between MSS voltages and power deposition in the He bath is still under investigation.

### Vibration measurements

**Fig. 5:**
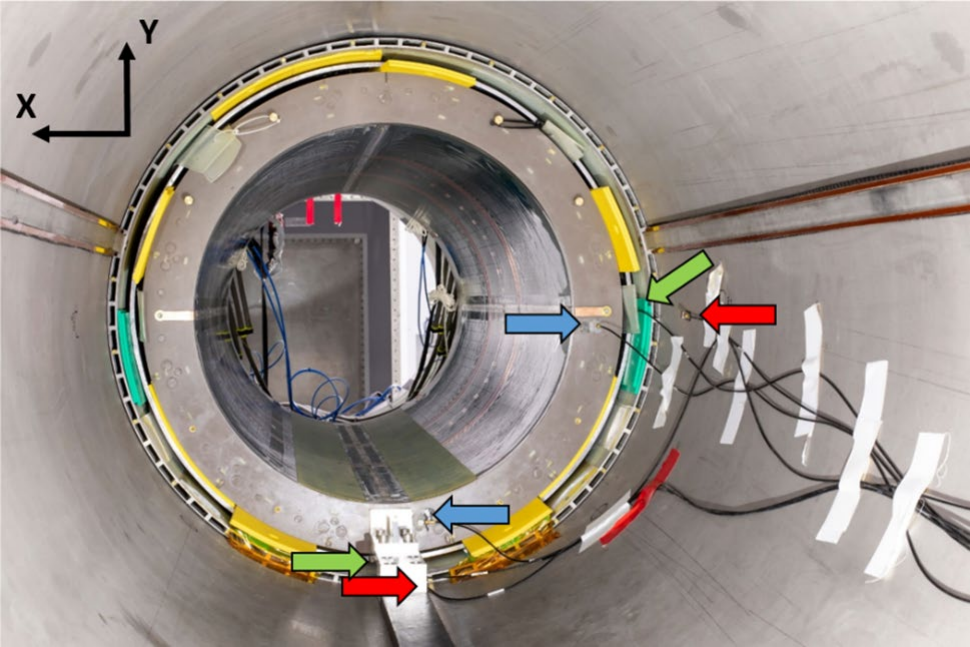
Vibration measurement setup. 6 mono-axial accelerometers were glued to measure accelerations either in the *X* or *Y* direction. The blue, green and red arrows point towards the locations of the accelerometers placed on the gradient coil, the lead tube and the cryostat, respectively. Accelerometers on the right and at the bottom measured vibrations along the *X* and *Y* directions, respectively

Vibrations are key to understand power deposition in the He bath, mechanical stress and potential image artefacts. In this context, measurements were performed with 6 mono-axial accelerometers (Brüel & Kjaër, Naerum, Denmark) connected to dedicated frontend and software. Because the main *B*_0_ field is along the *Z* direction, main forces operate along the *X* and *Y* directions so that the accelerometers were arranged to characterize accelerations along those axes. Figure 5 shows the setup: two were placed on the flange of the gradient coil, two on the lead tube and two on the cryostat (bore tube).

**Fig. 6:**
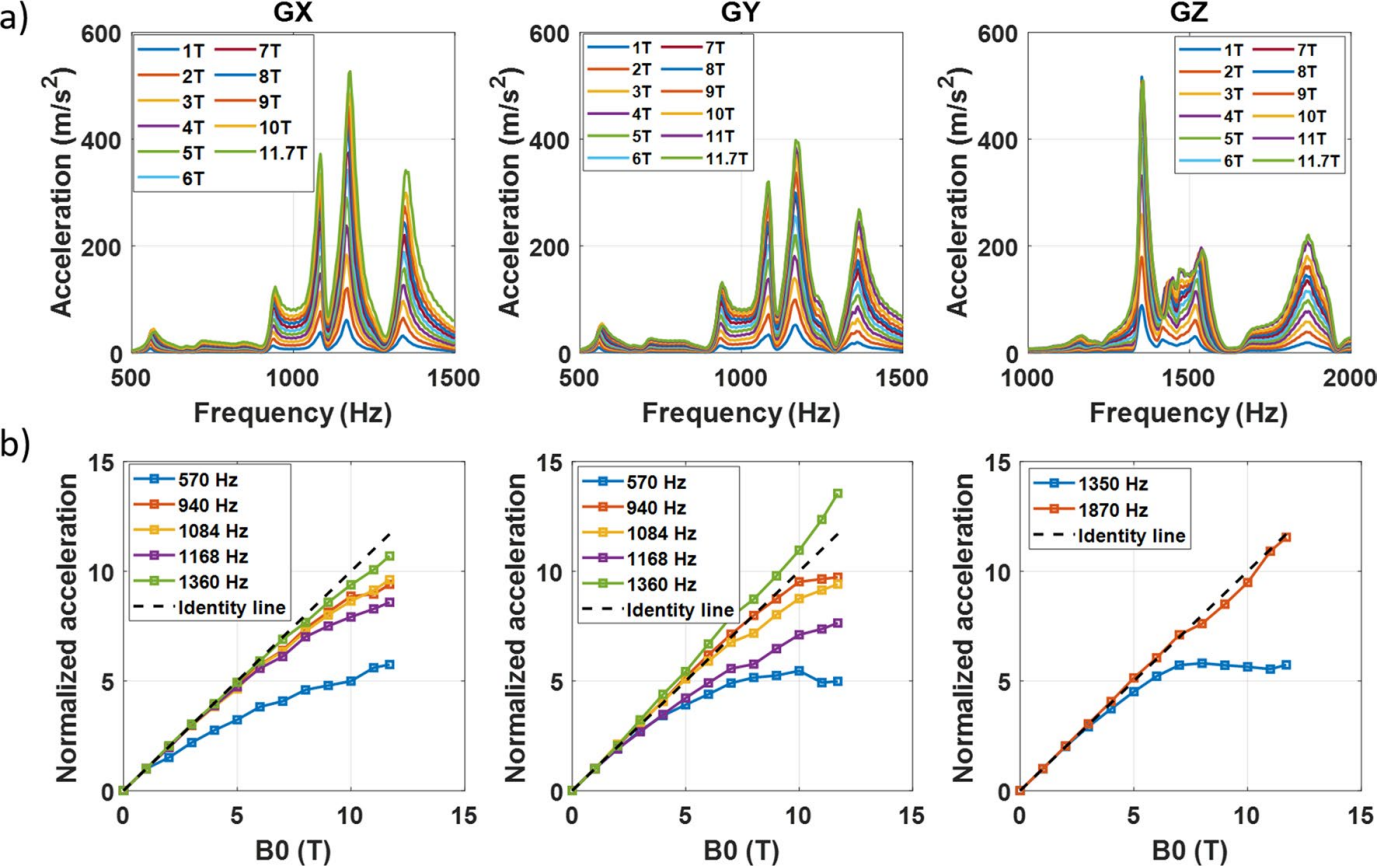
Vibration measurement results without lead tube (*G* = 1 mT/m). From left to right, the results for the *X-*, *Y-* and *Z*-axes are provided. Top row: acceleration versus frequency and different field strengths (different colors). A zoom on the main peaks is provided. Bottom row: acceleration peak heights for different resonance frequencies, normalized to the values obtained at 1 T. The dashed line corresponds to the linear trend

Measurements were performed with linear frequency sweeps over the 0–3 kHz range in 2 min. They were repeated at different gradient strengths (0.1, 1 and 5 mT/m) to reveal good linearity of the vibrations with respect to gradient excitation. The frequency output likewise was confirmed to be equal to the input frequency, in agreement with a linear theory. Vibrations were recorded from 0 to 11.7 T in steps of 1 T during ramp-up to study vibration behavior versus main *B*_0_ field. The results are reported in Fig. 6 (without lead tube) for the three gradient axes and at 1 mT/m gradient strength. Accelerations up to 50 g can be visualized so that at maximum gradient strength, over 1000 g accelerations and forces could be obtained. When converted to displacements, such values can lead to a couple hundreds of µm, advocating caution when some particular frequencies are used for imaging at ultra-high resolution. In this scenario, the errors and artefacts engendered will depend on the frequency spectrum of the MR sequence. An echo-spacing of 0.37 ms in EPI for instance yields slowly decreasing odd order harmonics whose first term at 1350 Hz (main peak on the GZ vibration spectrum) yields an amplitude of ~ 1.2 times the gradient intensity (*G*_max_), the exact number depending on the ramp durations. Taking 500 m/s^2^ the peak acceleration measured at 11.7 T on the *Z*-axis at 1 mT/m and at 1350 Hz, the displacement induced at this frequency can be assessed with $d={1.2\times G}_{\mathrm{max}}\times 500/{\upomega }^{2}$, with *G*_max_ in mT/m and *ω* the angular frequency. For a gradient intensity of 20 mT/m, one obtains 167 µm. Although this echo-spacing is not realistic for EPI and high resolution applications with the SC72 gradient specifications, more powerful gradients, yet with their own vibration spectra, can more aggressively hit these frequencies. Spatial distortions can be problematic, but large vibrations in addition can alter the field behavior for which spins are sensitive (see section on field monitoring). Vibrations in general increase with main field strength B_0_, as expected. To study more in depth their behavior, the height of the main resonance peaks, normalized to the one obtained at 1 T, is plotted versus the B_0_ field (bottom row of Fig. 6). Aside from one exception for the *Y*-axis (1360 Hz resonance), resonance amplitudes grow linearly with B_0_ field strength only in the worst case. Interestingly, some, e.g. the 1350 Hz resonance on the *Z*-axis and the 570 Hz resonance on the *Y*-axis, reach a plateau, i.e. vibrations no longer grow with the main field beyond a certain limit.

Given the good linearity of the system with respect to gradient excitation, a simple harmonic oscillator model around a resonance can be attempted to gain further insight. If $u$ denotes the displacement of the oscillator (corresponding to the location of the accelerometer), then it obeys the following second order differential equation: $\ddot{u}+\kappa \dot{u}+{\omega }_{0}^{2}u=\frac{F(t)}{m}$, where F is the Lorentz force proportional to $G\left(t\right){\times B}_{0}$ (itself proportional to current), ${\omega }_{0}$ is the resonance frequency and κ is the damping coefficient. If *G*(*t*) varies sinusoidally, at resonance, in the steady state and provided the Q factor is sufficiently high, the peak amplitude is ${u}_{\mathrm{max}}\propto \frac{{G}_{\mathrm{max}}{B}_{0}}{\kappa }$, where the linearity of this result versus gradient strength (*G*_max_) indeed could be confirmed experimentally. Within the limits of this simplified model, this suggests for the plateauing resonances that $\kappa \propto {\kappa }_{0}+\alpha {B}_{0}$, with ${\kappa }_{0}$ and $\alpha$ frequency-dependent constants, at least for the range of B_0_ fields investigated. Earlier work suggested instead damping of the form $\kappa \propto {\kappa }_{0}+\alpha {B}_{0}^{2}$ (so-called Lorentz damping) [23]. One possibility is the consideration that gradient amplifiers ideally are current sources which aim at maintaining desired currents despite eddy-currents. Of course, the intuition provided by the model above breaks down as soon as resonance peaks get mixed. Regardless of the underlying physical mechanism and its complexity, it remains of practical value that the height of the resonance peaks here increases linearly with *B*_0_ only in the worst case (one supra-linear exception on the *Y*-axis). This is an encouraging result for MR exploitation at UHF where vibrations, and hence also sound pressure levels, remain relatively under control as field strengths continue growing.

### Acoustic measurements

**Fig. 7:**
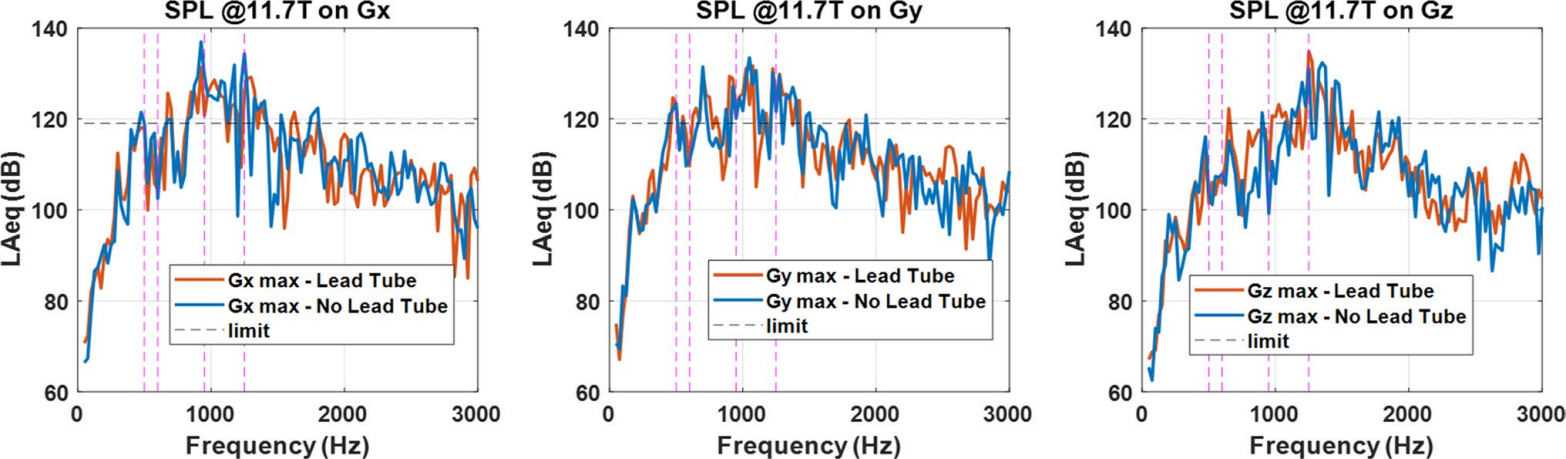
Sound pressure level (LAeq) measurement results at 11.7 T and with the service coil. Left to right: *X*, *Y* and *Z* gradient coil axis. The horizontal dashed line corresponds to the current limit imposed at NeuroSpin for volunteers. The vertical dashed lines bound two forbidden zones where echo-spacings in EPI are disabled in software

Sound Pressure Level (SPL) measurements were performed with a Rion NA-28 (Rion co, Tokyo, Japan) sound meter placed inside the service coil (16 rung shielded birdcage coil (QED, Mayfield Village, Ohio, USA)) at iso-center, at fixed gradient frequency (every 25 Hz) over the 0–3 kHz range (as representative of the range covered by MR sequences), at 100% duty cycle and at maximum gradient amplitude allowed by the hardware. Additional restrictions were imposed in some frequency intervals following recommendations from Siemens Healthineers, to prevent any damage of the gradient coil or cables. The results in A-weighted equivalent continuous sound levels (LAeq) are presented in Fig. 7. The horizontal dashed line represents the current 119 dB imposed by NeuroSpin for studies on human volunteers, assuming a conservative 20 dB sound insulation enabled by ear protection in order not to exceed the 99 dB IEC 60601-2-33 limit perceived by subjects. The vertical lines bound two «forbidden» zones centered around 550 and 1100 Hz respectively, where gradient mechanical resonances can affect image quality and damage the gradient coil (see vibration spectra of Fig. 6). Echo-spacings tuned to these zones in EPI sequences are normally disabled. Considering the 100% duty cycle and the zones where one normally does not operate directly in EPI, the measurement results are promising and suggest that tweaking the parameters of the MR sequences shall fulfill the current limits. Regarding the differences with and without lead tube, no clear benefit of one scenario versus the other could be identified.

### Field monitoring

**Fig. 8:**
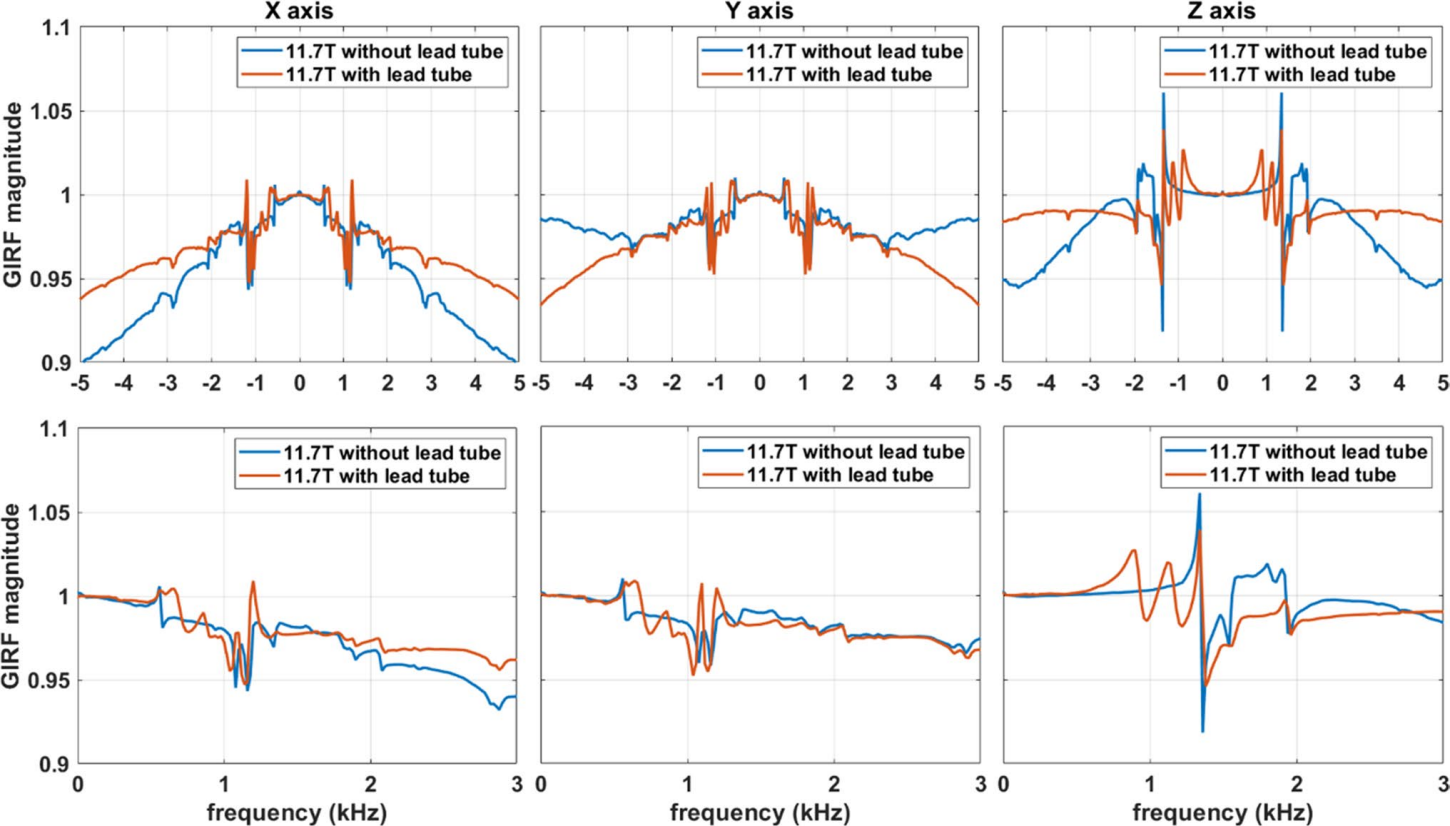
GIRF (first-order terms) spectra acquired at 11.7 T without and with lead tube (top row: − 5 to + 5 kHz interval, bottom row: zoom on the 0–3 kHz interval). The lead tube on the *Z*-axis led to unwanted peaks in the ~ 900–1150 Hz region. Removing the lead tube suppressed the peaks in that region but boosted some peaks at higher frequencies

Strong vibrations can be the source of hardware damage but also of image artefacts. The AROMA project (https://aroma-h2020.com/) is a H2020 European project gathering CEA, the University of Glasgow, the University of Maastricht, the German Center for Neurodegenerative diseases (DZNE), ETH Zürich and Skope (Skope MRT, Zürich, Switzerland). Its fundamental goal is to develop the pillar methodologies enabling optimal exploitation of the 11.7 T scanner. In this context, Skope MRT delivered to CEA as soon as possible in the project a clip on field camera destined for real-time field monitoring in vivo as well as field quality control. After proper positioning of the field probes to reconstruct the spatiotemporal field distribution up to the third harmonics of the spherical harmonics decomposition, characterization of the field dynamics was conducted with Gradient Impulse Response Function (GIRF) measurements [24]. The sequence consisted of repeating gradient blips, measuring the temporal field response and performing a Fourier transform with various settings to accurately cover a wide frequency range. The blips were short triangular waveforms played consecutively along the *X*, *Y* and *Z* gradient axes. Twelve blips were played on each axis with a slew rate of 180 mT/m/ms, duration increasing from 100 to 320 µs and a TR of 1 s. 50 averages were acquired to increase the SNR, resulting in a total acquisition time of 30 min. The self (first order) terms are displayed in Fig. 8. Such characterizations were first performed with the lead tube. Peaks clearly corresponding to mechanical resonances of the gradient coil could be identified. Despite the presence of some peaks on the *X*- and *Y-*axes, experimentally no major problem has been perceived so far for those axes at 11.7 T.

**Fig. 9:**
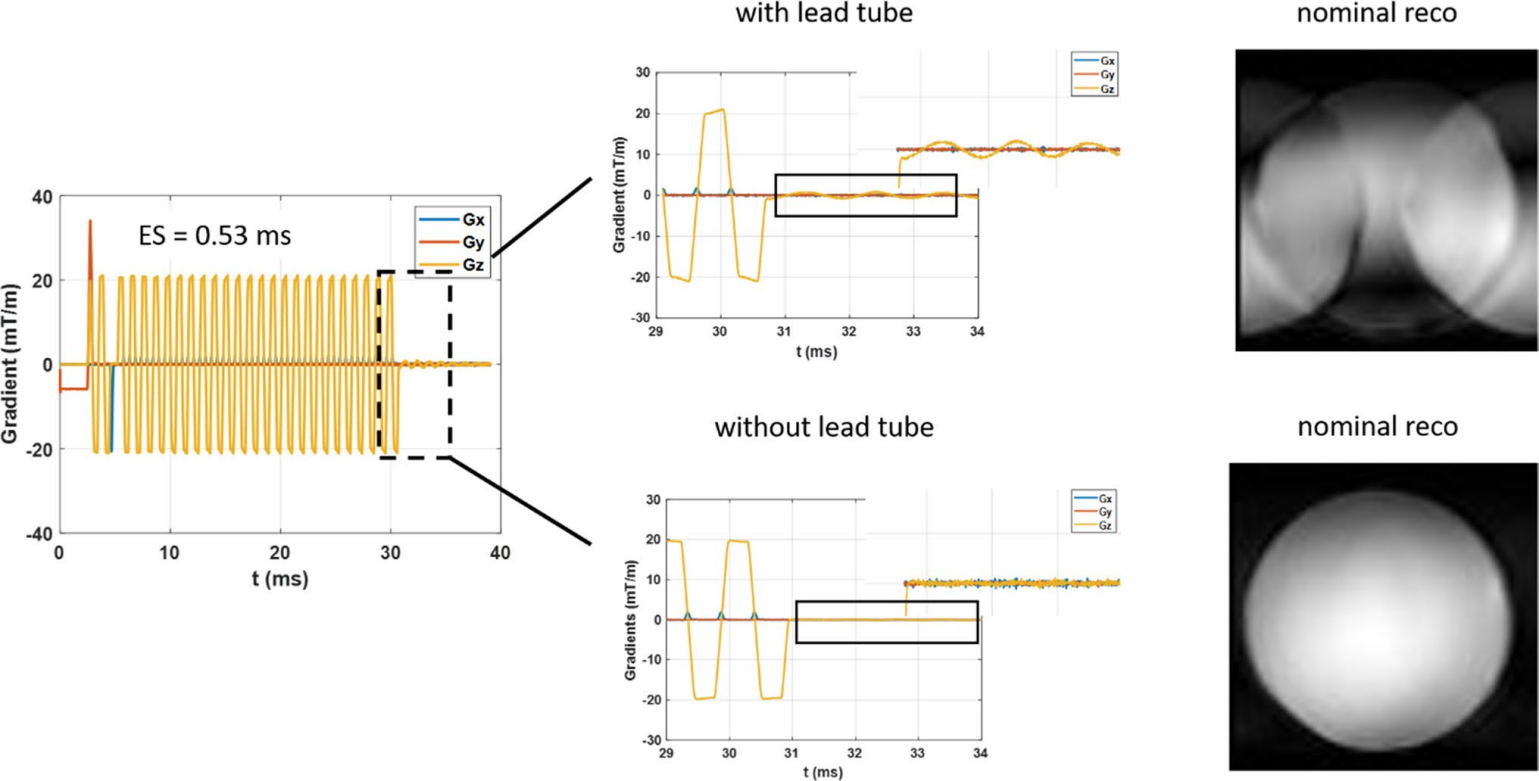
EPI gradient waveforms acquired with a Skope field camera at 11.7 T. As suggested by the GIRF spectrum, this particular echo-spacing engendered field distortions and oscillations in the presence of the lead tube, ultimately leading to strong ghosting artefacts. Removing the lead tube and repeating the exact same test revealed much more faithful gradient waveforms and better image quality with the standard image reconstruction pipeline

The peaks on the *Z*-axis, however, warranted further investigations. Figure 9 reports a gradient field measurement with an EPI sequence with the lead tube and whose echo-spacing (ES = 0.53 ms) excites directly (first harmonic) the frequency of 943 Hz. It shows that despite preemphasis and scanner calibration, the EPI-plateaus are not flat (~ 5% gradual change over the plateau) and oscillations occur at the end of the readout train (a signature of eddy-currents carried by vibrations). Ultimately, the field distortions led to strong ghosting artefacts when reconstructing the images with the standard pipeline. Further modelling (spherical harmonics decomposition of the field induced by eddy-currents) showed that the lead tube vibrations and eddy currents were responsible for the 900–1150 Hz peaks in the GIRF spectrum and the corresponding field distortions. As a result, after verifying the safe power depositions in the magnet, it was finally decided to remove the lead tube and repeat the measurements. The same figure shows in this situation more faithful behavior of the gradient waveform (flatter plateaus, no oscillations) with the same identical protocol, leading to a great reduction of the ghosting artefact, consistent with the disappearance of the 900–1150 Hz peaks without the lead tube in the GIRF *Z*-axis spectrum (Fig. 8). The same spectrum without the lead tube, on the other hand, still reveals an important, amplified, peak at 1350 Hz. This resonance, also visible in the cryogenics and vibration spectra, is known to arise from the strongest vibration mode of the gradient coil (Fig. 6). It is clear that stronger fields amplify the interactions between the gradient coil and the magnet, and can lead to field perturbations detrimental to imaging. Ways to decouple the two are currently under investigation. Although some vibration peaks of Fig. 6 seem to correspond to peaks in the cryogenic and GIRF spectra, it appears not straightforward to correlate their heights so that most likely the nature of the mode plays an important role as well. Finally, given the linear spatial variation of the field distortions seen in Fig. 9, one could intuit equal disturbance on the currents circulating in the gradient coil due to changes of impedance seen by the Gradient Power Amplifiers (GPA) [23]. Such disturbances indeed could be visualized on sensors measuring those currents directly on the GPA. With arbitrary waveform generation in an MR sequence, active compensation succeeded in cancelling to a large extent the current deviations. For reasons that remain to be determined, such measures yet were not enough to capture entirely the field perturbations.

### First images

**Fig. 10:**
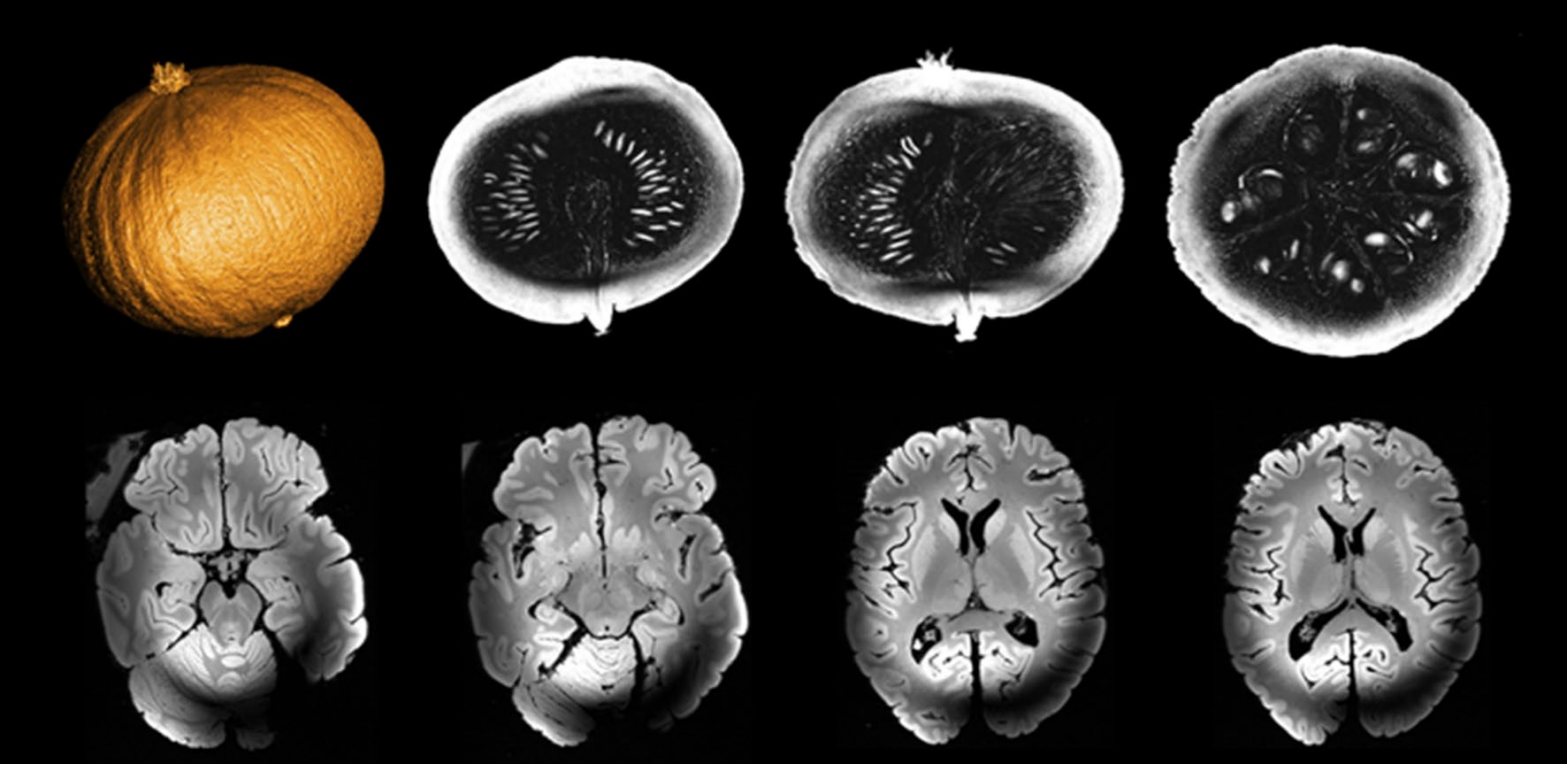
First images acquired on a pumpkin (top row) and ex-vivo brain (bottom row) at 11.7 T with the service volume coil (October 2021). 3D-GRE sequence parameters were: TR = 20 ms, TE = 1.8/2.5 ms (pumpkin/brain), 4 averages, 512 × 512 × 512 matrix

After ensuring that gradient activity would not pose a serious risk to the magnet, first images were acquired on a pumpkin and on an ex-vivo brain at 11.7 T in 2021 [17]. Figure 10 shows different slices (axial, coronal and sagittal on the pumpkin, only axial for the ex-vivo brain) acquired with a 3D GRE sequence at 0.4 mm isotropic resolution with the service volume coil. The RF field inhomogeneity artefact is clearly visible on the ex vivo acquisition and is inherent to the use of high fields and volume coils. Those acquisitions were a milestone in the history of the project confirming that Iseult was no longer just a magnet but had become an MRI machine. More quantitative measurements followed. The same (traveling) spherical phantom was scanned at 3 T, 7 T and 11.7 T at NeuroSpin CEA, at 7 T and 9.4 T at the University of Maastricht, and finally at 7 T and 10.5 T at the Center for Magnetic Resonance Research of the University of Minnesota [9]. SNR measurements at the center of the phantom were performed in quasi-identical conditions (phantom, positioning, MR protocol, temperature and volume coil). Results revealed an SNR trend proportional to ${B}_{0}^{1.94}$, in good agreement with theory, confirming this time quantitatively that Iseult delivers its MR potential.

**Fig. 11.**
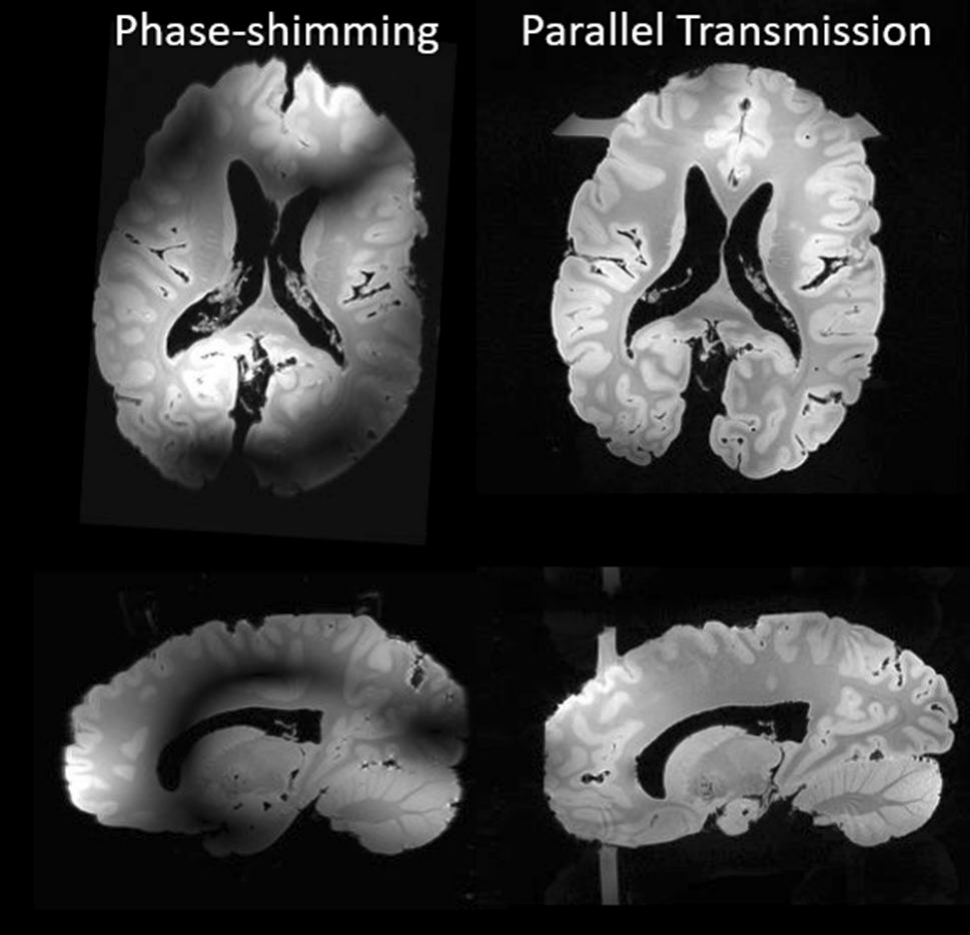
3D-GRE images acquired with the Iseult pTx RF coil on an ex vivo brain, with phase-shimming (left) and *k*_T_-points (right). Axial (top) and sagittal (bottom) slices are shown. The horizontal and vertical bars in the axial and sagittal views respectively, and for the *k*_T_-point excitation, result from the excitation of the fixation bar of the ex-vivo brain in its container

Figure 11 finally illustrates first parallel transmission tests performed with the home-made Iseult RF coil on an ex vivo brain [25]. The result is a 3D GRE acquisition (0.7 mm isotropic resolution, TR = 30 ms, TE = 4.6 ms, FA = 10°, TA = 5 min 30 s, iPAT = 2 × 2). The coil currently combines 15 transceiver and 17 receive-only elements and has a minimized outer diameter (27 cm) to fit in a local B_0_-shim multi-coil array. The elements are geometrically decoupled via resonant inductive decoupling elements. The coil features two rows of alternate loops and small dipoles (in fact air–gap center-fed microstrips), and a patch at the top of the head. Because only 8 RF amplifiers are for the moment available, the coil operates in a 8Tx-32Rx configuration where 14 transceive elements are coupled in pairs to connect to 7 amplifiers while the last element is connected alone. The combined use of the coil with *k*_T_-points parallel transmission pulse design [26] here allowed mitigating the RF field inhomogeneity problem at 11.7 T while, unsurprisingly, phase-shimming did not provide enough degrees of freedom. The horizontal and vertical “bars” in the axial and sagittal views, respectively, are caused by the fixation bar of the ex-vivo brain in its container, which returns some signal with the more broadband *k*_T_-point excitation.

## Discussion and conclusion

The commissioning of the Iseult 11.7 T WB MRI now is nearly complete and opens the door for exciting MR exploration of the human brain. Measurements to characterize the gradient–magnet interactions took nearly two years but were naturally slowed down (or even interrupted) due to the COVID pandemics. To our knowledge, some data and experience acquired with these test campaigns are unique and we hope they will contribute to advance knowledge and technology in this ultra-high-field realm. Yet, given the complexity of the system and the many factors potentially affecting the field behavior, it is too premature to extrapolate our results to other MR systems. Here two different scenarios were investigated, with and without a lead tube surrounding the gradient coil. Aside from an apparent residual mechanical coupling not taken into account in the model, the power deposition experimental data acquired on Iseult on the *Z*-axis seem to validate the magnet protection concept for which the lead tube was designed and installed [21]. After power depositions in its presence were verified to be safe, the lead tube yet was finally removed to suppress some unwanted field behavior and image artefacts. Because some design choices could be irreversible, it also appears fundamental that for risk minimization in such UHF endeavors, more efforts should be made to develop more complete modeling software tools to predict the field behavior. To our knowledge again, current methods describe electromagnetic interactions between different shells, their induced vibrations, but lack taking into account their mechanical coupling to reduce the, already great, complexity of the problem.

Although these interactions may not be as critical at 7 T, their amplification at 11.7 T and beyond can become problematic and warrants further investigations. It is clear that the source of the remaining peak at 1350 Hz on the GIRF spectrum arises from a mechanical resonance of the gradient coil (visible on Fig. 6). Interestingly, the vibration data at that frequency reveal a plateau behavior where vibrations no longer increase with field strength beyond approximately 7 T. The latter could be perceived as positive in terms of imaging performance and acoustics for explorations beyond 7 T, but one also is naturally led to wonder whether these phenomena (more intense peaks in the GIRF spectra and vibration plateau behaviors) are related. Current evidence (data not shown) indeed suggests that the peaks in the GIRF spectra may increase supra-linearly with B_0_ (according to measurements performed at 7 T on the same system). To fully take advantage of the large portfolio of MR sequences and their parametrizations, unless the problem is solved at the source, adding more restrictions on the MR sequence spectrum or having more recourse to non-Cartesian reconstructions based on knowledge of the field dynamics [27] would likely be necessary. Testing a new configuration currently necessitates ramping down the field. Although up to now the system has been ramped up and down a dozen times, this is not without risk and the number of cycles should be minimized. For this reason, future tests will likely aim at identifying the cause of the remaining peak by ramping the system at 7 T with modifications of the setup and performing field monitoring at that field strength (imaging not being possible at 300 MHz due to incompatible electronics). Magnet safety system voltage measurements have also appeared to be very sensitive to the experimental conditions and can be performed quickly and at any field strength to diagnose safely possible improvements or deteriorations. Again modeling imposes itself as a wise strategy to minimize risks and experimental efforts for the future.

Field monitoring [28] has been an invaluable tool to understand and troubleshoot our system. While first MR images acquired with the lead tube appeared flawless, small changes in some protocols suddenly revealed subtle artefacts. Ultimately, in the worst case strong ghosting artefacts appeared at particular echo-spacings in EPI. Unless they are textbook cases, going from image artefacts to the field dynamics would be incredibly more cumbersome and time-consuming. Field monitoring allows the user to picture the field dynamics in a matter of seconds and understand the root cause, field-wise, of the problem. GIRF measurements [24] finally provide a nearly full picture of the field behavior over a broad frequency range, whereas testing particular sequences and protocols may miss by chance certain field resonances. This will likely be used as a quality control tool in the future to detect possible abnormal changes of the field dynamics over time. Gradient–magnet interactions increase with the development of more powerful magnets and gradient coils [29, 30]. Field monitoring appears to us today as a quasi-necessity to secure the corresponding large investments. In our experience, monitoring the currents injected directly in the gradient coils was not enough to capture the full extent of the field behavior.

The next important milestone in the life of Iseult is the authorization to scan human subjects. Although no significant adverse effects have been shown at 10.5 T on volunteers [31], caution remains advised as some effects do appear detectable while their relations to field strength and duration of exposure are presumably unknown [32, 33]. As a result, more experiments and data for exploitation at 11.7 T or higher are warranted. These questions have also been the subject of an on-going enterprise carried out at NeuroSpin and first submission to the French regulatory body occurred end of 2022. Provided the authorization is granted, first in vivo experiments on adult volunteers are planned for 2023.

## Data Availability

Data is available upon reasonable request.
